# Emergency hospital admissions, prognosis, and population mortality in Norway during the first wave of the Covid-19 epidemic

**DOI:** 10.1177/14034948221082959

**Published:** 2022-05-12

**Authors:** Henriette C. Jodal, Frederik E. Juul, Ishita Barua, Michael Bretthauer, Mette Kalager, Magnus Løberg, Louise Emilsson

**Affiliations:** 1 Clinical Effectiveness Research Group, University of Oslo and Oslo University Hospital, Norway; 2 Department of General Practice, University of Oslo, Norway; 3 Vårdcentralen Värmlands Nysäter & Center for Clinical Research, Region Värmland, Sweden; 4 School of Medical Science, University of Örebro, Sweden; 5 Department of Medical Epidemiology and Biostatistics, Karolinska Institutet, Sweden

**Keywords:** Covid-19, public health, health policies, emergency medicine, internal medicine, surgery

## Abstract

**Background:**

During the first wave of the Covid-19 epidemic, a national lockdown was established in Norway, and inhabitants were asked to contact healthcare only if absolutely necessary. We investigated hospital admissions and mortality due to non-Covid-19 disease during the lockdown compared to previous years.

**Methods:**

We compared the number of emergency admissions and in-hospital fatality for diagnoses probably unaffected (acute myocardial infarction, acute abdominal conditions, cerebrovascular diseases) and affected by the lockdown (infections, injuries) in the South-Eastern Health Region of Norway during weeks 12–22, 2020, compared to the mean of the same period in the years 2017–2019. We also compared population mortality March–May 2020, to the mean of the same period in years 2017–2019.

**Results:**

A total of 280,043 emergency admissions were observed; 20,911 admissions probably unaffected, and 30,905 admissions probably affected by the lockdown. Admissions due to diagnoses probably unaffected was reduced by 12% (95% confidence interval (CI) 9–15%), compared to 2017–2019. Admissions for diagnoses probably affected was reduced by 30% (95% CI 28–32%). There was a 34% reduction in in-hospital fatality due to acute myocardial infarction (95% CI 4–56%), 19% due to infections (95% CI 1–33%), and no change for the other diagnoses, compared to 2017–2019. The risk of in-hospital mortality to total mortality was lower for acute myocardial infarction (relative risk 0.85, 95% CI 0.73–0.99) and injuries (relative risk 0.83, 95% CI 0.70–0.98).

**Conclusions:**

**Even though fewer patients were admitted to hospital, there was no increase in in-hospital fatality or population mortality, indicating that those who were most in need still received adequate care.**

## Background

Covid-19 was declared a pandemic by the World Health Organization (WHO) on 11 March 2020 [1]. The following day, the first Covid-19-related death occurred in Norway [2], and the Norwegian government implemented the strongest public measures since World War 2 to fight the epidemic: domestic and international travel restrictions [3], home office for everyone when possible [3,4], schools and day cares closed [3], and organized sports, cultural events, and other gatherings were restricted (Table S1) [3]. All inhabitants were asked to stay at home [3,4], and to only contact healthcare if absolutely necessary [4]. Elective surgery was postponed, and consultations for patients with chronic diseases were performed through phone and video [4].

Shortly after, the first reports of a worrisome decline of the number of patients seeking healthcare were observed, including a reduction in emergency contacts and cancer diagnostics [5,6]. Several reports on the decrease of non-Covid-19-related hospital admissions have since been published internationally, with decreased admissions due to stroke [7–11], acute myocardial infarction [12–16], and appendicitis [17–19]. However, these reports are hampered by focus on one disease group only [7–15,17,18,20–23], small sample size [7,9–15,17–20,23], or lack of valid comparison groups [7–10,12–15,17,19–25].

We investigated non-Covid-19 hospital admissions, in-hospital fatality, and mortality for diseases which require urgent and timely healthcare to avoid severe disability or death during the first lockdown in the largest health region of Norway, adjusting for seasonal and temporal trends by comparison with corresponding periods in the three previous years, as well as the weeks preceding the epidemic. The diseases were divided into two categories, for which we expected different direct effects of the lockdown: diseases that are probably affected by lockdown, and diseases that are probably unaffected by lockdown.

## Methods

### Study setting

Norway has a single-payer public healthcare system. The public hospital system consists of four health regions, of which the South-Eastern Health Region comprises 56% of the total population of Norway. From 1 January 2017 to 1 January 2020, the population of the South-Eastern Health Region increased by 2.7%, from 2.95 to 3.03 million people. The number of people above 85 years increased by 1.1%, and the median age increased from 39 years in years 2017–2019 to 40 years in year 2020. As these increases are small, we did not adjust for population changes in this study.

The national lockdown was implemented on 12 March 2020, with gradual opening from 27 April to 15 June (Table S1). Some minor restrictions were further continued – for example, number of people allowed at cultural events.

### Study design

#### Hospital admissions

We retrieved information on weekly hospital admissions from the electronic health records of all eight somatic care health trusts in the South-Eastern Health Region of Norway. Information was retrieved for admissions during weeks 2–22 (January through May) of the years 2017 through 2020. We retrieved data on the number of emergency admissions regardless of diagnosis, as well as the number of admissions for patients discharged with the following selected International Classification of Diseases tenth revision (ICD-10) codes usually requiring emergency admissions: acute myocardial infarction (I21), acute abdominal conditions (K35: acute appendicitis; K56: paralytic ileus and intestinal obstruction without hernia), cerebrovascular disease (G45: transient cerebral ischaemic attacks and related syndromes; I60–I64: nontraumatic intracranial haemorrhage and cerebral infarction), infections not including Covid-19 (G00–G05: meningitis and encephalitis; J9–J11: influenza; J13–J15: bacterial pneumonia; J16–J18: other pneumonia), and injuries (S12, S22, S32, S42, S52, S62, S72, S82, S92, T02: bone fractures; S02, S05, S06-08: head injuries). The number of admissions were stratified by sex, age group (0–44 years, 45–64 years, 65 years and older) and vital status at discharge (dead or alive). All data on hospital admissions were stratified by week using the International Organization of Standardization (ISO) 8601-week numbering, in which the week starts with Monday, and week 1 of the year is the week with the year’s first Thursday.

We divided the abovementioned diagnosis categories into two groups:

1) Diagnoses probably unaffected by the lockdown: emergency admissions that need emergency healthcare and we expect to occur at the same frequency regardless of Covid-19 mitigation measures; acute myocardial infarction, acute abdominal conditions, and cerebrovascular disease.
2) Diagnoses probably affected by the lockdown: emergency admissions that need emergency healthcare but where we expect the frequency of disease to be affected by Covid-19 mitigation measures; infections and injuries.

We performed sensitivity analyses for cerebrovascular disease excluding transient cerebral attacks and related syndromes (ICD-10 G45), as this diagnosis does not cause mortality in the short-term.

#### Population mortality

We retrieved publicly available data on the total number of deaths and number of in-hospital deaths for the whole of Norway for the period 1 March–31 May (approximately week 10 through 22) in years 2017 to 2020, stratified by cause of death, from the Norwegian Cause of Death Registry.

### Analyses

#### Hospital admissions

We analysed hospital admissions data in weeks 12–22 – that is, the weeks following the outbreak of the Covid-19 epidemic and the implementation of mitigation measures in Norway, including lockdown [2,3]. We also performed analyses for the period preceding the outbreak (week 2–10) (Supplementary material).

We compared the two weekly periods for year 2020 to the same periods in each of the non-epidemic years (2017, 2018, and 2019), and to the mean of the same periods of the non-epidemic years. We calculated absolute differences (AD) and relative risks (RR) of the number of admissions in each year, compared to the mean of the non-epidemic years. The same calculations were performed for readmissions and for in-hospital fatality. We calculated the mean number of days in hospital for each diagnosis group and compared each year, respectively, to the mean of the non-epidemic years, using Students’ *t*-test.

#### Population mortality

Weekly data from the Cause of Death Registry were not available. Thus, we analysed cumulative mortality for the period 1 March to 31 May (approximately week 10 through 22). We compared this period of the year 2020 to the same period in each of the non-epidemic years (2017, 2018, and 2019), and to the mean of the same period of the non-epidemic years. We calculated ADs and mortality ratios (MR) for cause-specific deaths for the following diagnoses: acute myocardial infarction, cerebrovascular disease, pneumonia, influenza, and injuries. The same calculations were performed for in-hospital mortality. Stratification on county or health region was not available when stratifying for death place.

To evaluate whether the distribution of deaths inside and outside hospital differs from previous years, we calculated the RR for in-hospital to total mortality for each cause of death, comparing the year 2020 to the mean of the non-epidemic years.

All calculations include 95% confidence intervals (CI). *P*-values less than 0.05 were considered statistically significant. All analyses were performed in Stata 16.1 (StataCorp, TX, USA).

### Endpoints

Our primary endpoints were change in number of hospital admissions and in-hospital fatality. Secondary endpoints were change in readmissions, number of days in hospital, and population mortality.

### Ethical approval

The study was approved by the Regional Ethics Committee of South-Eastern Norway (no. 148608), and the Data Protection Offices at all included health trusts. Individual informed consent was waived by the Regional Ethics Committee of South-Eastern Norway due to the registry-based design of the study.

## Results

### Emergency admissions

**Figure 1.**
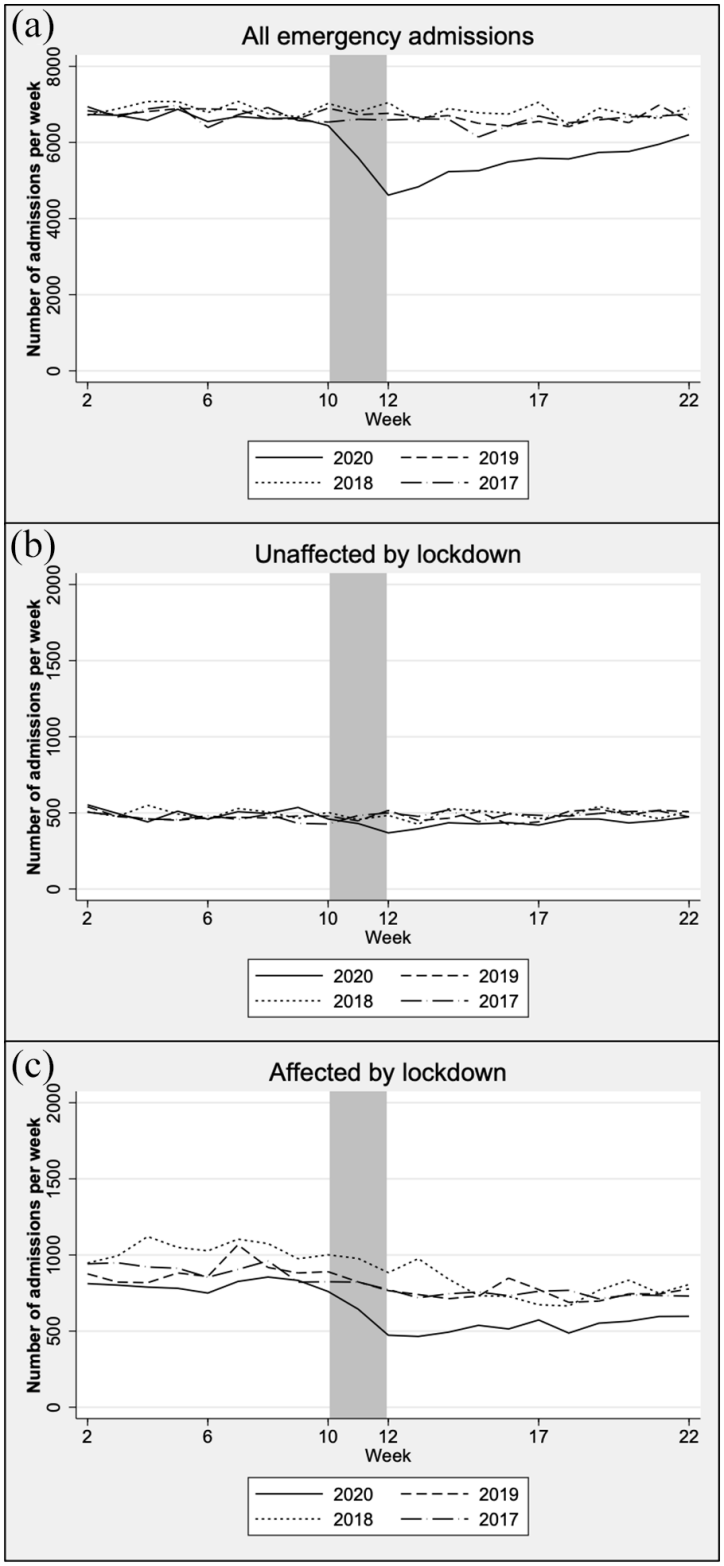
Weekly number of (a) all emergency admissions, (b) diagnoses probably unaffected by lockdown, and (c) diagnoses probably affected by lockdown. The grey area marks the implementation of the mitigation measures.

A total of 280,043 emergency admissions were registered in weeks 12–22 of years 2017–2020, which includes the lockdown period in 2020. On average, there were 5477 weekly emergency admissions in weeks 12–22, 2020 (range 4616–6202) and 6661 weekly emergency admissions in the same period the previous years (range 6144–7058) – an 18% decrease (RR 0.82, 95% CI 0.81–0.83) (Tables I, Table S3, Figure 1(a)).

**Table I.** Changes in admissions during the first wave of Covid-19. AD and RR are calculated with the mean of 2017–2019 as the reference, compared to 2020.

Diagnosis group	Diagnosis category	Year	AD	CI (95%)	RR	CI (95%)
Unaffected by lockdown	Acute myocardial infarction	Mean 2017–2019	0		1.00	
		2020	−36	(−45 to –27)	0.82	(0.78–0.86)
	Acute abdominal conditions	Mean 2017–2019	0		1.00	
		2020	−2	(−9 to 5)	0.98	(0.92–1.05)
	Cerebrovascular disease	Mean 2017–2019	0		1.00	
		2020	−18	(−27 to −10)	0.90	(0.85–0.95)
Affected by lockdown	Infections	Mean 2017–2019	0		1.00	
		2020	−133	(−143 to −125)	0.51	(0.49–0.54)
	Injuries	Mean 2017–2019	0		1.00	
		2020	−94	(−107 to −80)	0.81	(0.78–0.83)

### Admissions unrelated to lockdown

**Figure 2.**
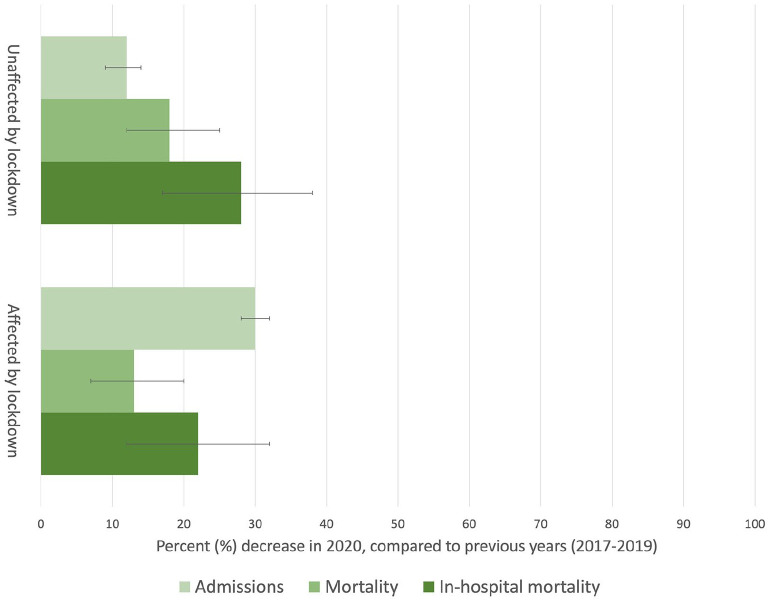
Percent (%) decrease in admissions, total population mortality, and in-hospital mortality. Admissions to hospital (light green), total population mortality (medium green), and in-hospital mortality (dark green) for diagnoses probably unaffected by lockdown and diagnoses probably affected by lockdown. The decrease is compared to the mean of the previous years (2017–2019).

A total of 20,911 emergency admissions were included in the diagnosis group where we did not expect change due to lockdown in weeks 12–22 in the years 2017–2020 (Table I). The number of admissions was 12% lower in 2020 than in previous years (RR 0.88, 95% CI 0.85–0.91) (Figures 1(b) and 2, Table S3).

Compared to the previous years, the number of admissions for acute myocardial infarction was 18% lower during weeks 12–22, 2020 (RR 0.82, 95% CI 0.78–0.86) (Table II, Table S4, Figure S1A). The reduction was similar for women and men (Table S5), but statistically significant only for patients older than 45 years (0–44 years: RR 0.89, 95% CI 0.65–1.20; 45–64 years: RR 0.80, 95% CI 0.73–0.88; ⩾65 years: RR 0.76, 95% CI 0.71–0.82) (Table S6). The number of admissions for acute abdominal conditions was not changed (RR 0.98, 95% CI 0.92–1.05) (Table II, Table S4, Figure S1B), irrespective of sex (Table S5) and age (Table S6). The number of admissions due to cerebrovascular disease was reduced by 10% (RR 0.90, 95% CI 0.85–0.95) (Table II, Table S4, Figure S1C), irrespective of sex (Table S5), but statistically significant only for patients older than 45 years (0–44 years: RR 0.77, 95% CI 0.58–1.02; 45–64 years: RR 0.85, 95% CI 0.74–0.98; ⩾65 years: RR 0.88, 95% CI 0.83–0.95) (Table S6).

**Table II.** Changes in in-hospital fatality during the first wave of Covid-19. AD and RR is calculated with the mean of 2017–2019 as the reference, compared to 2020.

Diagnosis group	Diagnosis category	Year	AD	CI (95%)	RR	CI (95%)
Unaffected by lockdown	Acute myocardial infarction	Mean 2017–2019	0		1.00	
		2020	−2	(−3 to 0)	0.66	(0.44–0.96)
	Acute abdominal conditions	Mean 2017–2019	0		1.00	
		2020	−1	(−1 to 0)	0.52	(0.22–1.06)
	Cerebrovascular disease	Mean 2017–2019	0		1.00	
		2020	1	(−1 to 4)	1.19	(0.94–1.50)
Affected by lockdown	Infections	Mean 2017–2019	0		1.00	
		2020	−3	(−5 to 0)	0.81	(0.67–0.99)
	Injuries	Mean 2017–2019	0		1.00	
		2020	−1	(−2 to 1)	0.87	(0.61–1.20)

A total of 608 in-hospital deaths occurred among patients admitted to hospital with a diagnosis probably unaffected by lockdown in weeks 12–22 of the years 2017–2020. Compared to the previous years, in-hospital fatality due to acute myocardial infarction was reduced by 34% during weeks 12–22, 2020 (RR 0.66, 95% CI 0.44–0.96) (Table III, Table S7, Figure 2), but unchanged for acute abdominal conditions (RR 0.52, 95% CI 0.22–1.06) and cerebrovascular disease (RR 1.19, 95% CI 0.94–1.50) (Table III, Table S7, Figure 2).

The mean length of hospital stay was slightly shorter for patients with cerebrovascular disease during weeks 12–22 in 2020 compared to previous years (0.67 days shorter, 95% CI −1.17 to −0.17), but not changed for any other patient group (Table S8). Forty percent fewer (RR 0.60, 95% CI 0.49–0.71) patients were readmitted within one week after an acute myocardial infarction during weeks 12–22 in 2020, while there was no change in readmission for the other patient groups (Table S9).

**Table III.** Total population mortality and in-hospital mortality. Selected causes corresponding to the groups unaffected by lockdown (acute myocardial infarction and cerebrovascular disease) and affected by lockdown (influenza, pneumonia, and injuries) in Norway 1 March–31 May 2020. MR is calculated with the mean of 2017–2019 as the reference, compared to 2020.

Diagnosis group	Death cause	Year	Total mortality				In-hospital mortality			
			No. of deaths	Lowest–highest	MR	95% CI	No. of deaths	Lowest–highest	MR	95% CI
Unaffected by lockdown	Acute myocardial infarction	Mean 2017–2019	495	(447–523)	1.00		195	(180–221)	1.00	
		2020	380	−	0.77	(0.68–0.86)	127	−	0.65	(0.53–0.79)
	Cerebrovascular disease	Mean 2017–2019	555	(542–572)	1.00		198	(187–217)	1.00	
		2020	529	−	0.95	(0.86–1.05)	178	−	0.90	(0.76–1.06)
Affected by lockdown	Influenza	Mean 2017–2019	83	(42–123)	1.00		38	(22–59)	1.00	
		2020	35	−	0.42	(0.29–0.60)	12	−	0.32	(0.16–0.58)
	Pneumonia	Mean 2017–2019	410	(367–456)	1.00		137	(124–150)	1.00	
		2020	302		0.74	(0.65–0.84)	193		0.75	(0.60–0.94)
	Injuries	Mean 2017–2019	473	(446–488)	1.00		156	(142–168)	1.00	
		2020	452	−	0.96	(0.86–1.06)	123	−	0.79	(0.64–0.97)

In sensitivity analysis excluding transient ischaemic attacks from cerebrovascular diseases, results were similar, except the reduction in admissions was statistically significant only for the age group 65 years and older (0–44 years: RR 0.76, 95% CI 0.56–1.03; 45–64 years: RR 0.88, 95% CI 0.75–1.02; ⩾65 years: RR 0.91, 95% CI 0.85–0.99).

### Admissions related to lockdown

A total of 30,905 admissions were included in the diagnosis group where we did expect change due to lockdown in weeks 12–22 of the years 2017–2020 (Table I). The total number of admissions was 30% lower (RR 0.70, 95% CI 0.68–0.72) in 2020 than in the previous years (Table S3, Figures 1(c) and 2).

Compared to previous years, the number of admissions due to infections was reduced by 49% during weeks 12–22, 2020 (RR 0.51, 95% CI 0.49–0.54) (Table II, Figure S2A), more in women than men (women: RR 0.44, 95% CI 0.40–0.48; men: RR 0.58, 95% CI 0.54–0.62) (Table S5), and in the age group 0–44 years (RR 0.38, 95% CI 0.31–0.45) (Table S6). The number of admissions due to injuries was reduced by 19% (RR 0.81, 95% CI 0.78–0.83) (Table II, Figure S2B), more in men than women (women: RR 0.85, 95% CI 0.73–0.81; men: RR 0.77, 95% CI 0.73–0.81) (Table S5), and less in the group 65 years and older (RR 0.94, 95% CI 0.89–0.99) (Table S6).

A total of 842 in-hospital deaths occurred among patients admitted to hospital with a diagnosis probably affected by lockdown in weeks 12–22 of the years 2017–2020. Compared to previous years, in-hospital fatality for patients admitted due to infections was reduced by 19% during weeks 12–22, 2020 (RR 0.81, 95% CI 0.67–0.99) (Table III, Table S7). For patients admitted for injuries, there was no change in in-hospital fatality (RR 0.87, 95% CI 0.60–1.20) (Table III, Table S7, Figure 2).

Compared to previous years, there was no change in length of hospital stay for neither injuries (0.02 days longer, 95% CI −0.25–0.29) nor infections (0.25 days shorter, 95% CI −1.02–0.51) during weeks 12–22, 2020 (Table S8). For patients admitted due to infections, readmission rate fell by 84% (RR 0.16, 95% CI 0.03–0.50), while it was similar for patients admitted for injuries (RR 0.92, 95% CI 0.64–1.29) (Table S9).

### Population mortality

Mortality due to acute myocardial infarction in Norway was 23% lower in March to May 2020 compared to the same period in years 2017–2019 (MR 0.77, 95% CI 0.68–0.86), 58% lower for influenza (MR 0.42, 95% CI 0.29–0.60), and 26% lower for pneumonia (MR 0.74, 95% CI 0.65–0.84) (Table III, Figure 2). There was no change in mortality due to cerebrovascular disease (MR 0.95, 95% CI 0.86–1.05) or injuries (MR 0.96, 95% CI 0.86–1.06). During the same time period, there was a reduction in in-hospital mortality due to acute myocardial infarction (MR 0.65, 95% CI 0.53–0.79), influenza (MR 0.32, 95% CI 0.16–0.58), pneumonia (MR 0.75, 95% CI 0.60–0.94), and injuries (MR 0.79, 95% CI 0.64–0.97), but not for cerebrovascular disease (MR 0.90, 95% CI 0.76–1.06) (Table III, Figure 2).

The risk of in-hospital mortality to total mortality was similar in year 2020 compared to the mean of the three previous years for cerebrovascular disease (RR 0.94, 95% CI 0.82–1.08), influenza (RR 0.76, 95% CI 0.47–1.22), and pneumonia (RR 1.02, 95% CI 0.86–1.22) (Table III, Figure 2). It was slightly decreased for acute myocardial infarction (RR 0.85, 95% CI 0.73–0.99) and injuries (RR 0.83, 95% CI 0.70–0.98) (Table III, Figure 2).

## Discussion

We found that emergency hospital admissions in South-Eastern Norway was 18% lower during the first Covid-19 lockdown as compared to the three previous years. Among patients with diagnoses that are probably unaffected by the lockdown, there was a 12% reduction in admissions in during the lockdown, and the reduction was confined to acute myocardial infarction and cerebrovascular diseases. For emergency patients with diagnoses that are probably affected by the lockdown – that is, infections and injuries – the reduction was 30%.

When frequency of hospital admissions is reduced, one would expect the admitted cases to be the most severe ones; hence, the proportion of severe and potentially life-threatening cases may be expected to increase. However, despite reduced hospital admissions, we did not observe any increase in in-hospital fatality compared to previous years, nor length of stay in hospital, nor readmission ratios.

It is unclear if the lower level of acute myocardial infarction admissions in 2020 is caused by the lockdown, since there was a decreasing trend also in the weeks preceding the Covid-19 outbreak (Table S4). There has indeed been a downwards trend in the number of admissions and deaths caused by acute myocardial infarction in Norway over the past 20 years [26], which is believed to be due to reduction in risk factors [27–29] and earlier identification of the disease [28].

The in-hospital mortality is slightly decreased during Covid-19 epidemic, which might indicate that more people died from acute myocardial infarctions outside hospital. However, at the time of data acquisition, the cause of death was still unknown in 5.6% of the deaths that occurred during the period of 1 March–1 May 2020. Thus, this slight decrease may be subject to change. While there is no similar declining trend in admissions for cerebrovascular disease, the risk of in-hospital mortality to total deaths remains the same, and there is thus no evidence that the reduction in hospital admissions has caused increased mortality at home.

When the first Covid-19 death occurred in Norway, strict mitigation measures were immediately implemented, including a stay-at-home order, and the closure of day cares and schools (Table S1) [3,4]. From halfway through our study period (week 17) these mitigation measures were gradually eased; however, with continued focus on distancing and hygiene, such as handwashing. This is reflected in the admission data, where the number of admissions due to infections remains low throughout the first wave of the Covid-19 epidemic (Figure S2A). For injuries, the decrease in admissions was most prominent early in the study period (Figure S2B), and normalized towards the end of the study period when the stay-at-home order ceased, and schools and day cares opened (Table S1).

The reduction in admissions due to acute myocardial infarction is worrisome if the reduction is due to individuals with chest pain refraining from seeking healthcare or not being admitted to hospital during the lockdown. However, we did not observe an increase in deaths due to acute myocardial infarction during this period, neither in-hospital or overall, as would be expected if individuals with acute myocardial infarctions did not receive appropriate care. The reduced number of admissions due to neurological disease was limited to the oldest age group when excluding transient ischaemic attacks. This adds to the findings in a smaller Norwegian study [11], where admissions due to cerebrovascular disease in only one of the health trusts studied here was included. Individuals experiencing transient ischaemic attacks not being admitted may be due to increased use of outpatient care, or that the individual did not seek healthcare. The latter might cause an increase in cerebrovascular events in the future, and this study is limited to the immediate consequences of disease.

This study is limited by the lack of long-term outcome data, more detailed information about disease severity, and information on chronic conditions to study the full impact of the lockdown. In addition, the study is limited by missing cause of death in 5.6% of the deaths reported 1 March–1 May 2020, as well as using mortality data from the full Norwegian population rather than only the same health region as the admissions data. The study is also limited by the inclusion of only one health region in Norway. However, this region has by far the largest population in Norway, and is also the region that was most affected by the first wave of Covid-19 [30]. Lastly, the categorization of diseases into probably affected and probably unaffected by lockdown may oversimplify the development of disease. Our definition of diseases probably affected by lockdown was based on direct causal relations (less mobility implies less accidents; less contact with others implies fewer infections); however, one could also include indirect relations – for example, the contribution of infections in the development of acute myocardial infarction [31,32].

In contrast to previous studies [7–19], we found a relatively small decrease in hospital admissions for patients with diagnoses that are probably unaffected by the lockdown: acute myocardial infarction, acute abdominal conditions, and cerebrovascular disease. A US study of non-Covid-19-related admissions during the epidemic [19] found that admission rates for diagnoses probably unaffected by lockdown declined less than other diagnoses, but the decline was larger than what we found. This may partly be explained by the differences in healthcare organization and social security benefits between the US and Norway. The same study also finds that the number of admissions due to infections remains lower longer than for diagnoses probably unaffected by lockdown, consistent with our findings. In-hospital mortality in several emergency admissions has only been studied in two previous publications [19,21], which both showed no difference in mortality. This is in line with our findings, which are additionally adjusted for seasonal and temporal trends.

## Conclusions

This is the first study to evaluate changes in non-Covid-19 emergency hospital admissions and death of diseases requiring timely and life-saving healthcare during the Covid-19 epidemic, adjusting for both seasonal and temporal trends. Even though fewer patients were admitted to hospital for these diseases in Norway, there was no increase in in-hospital fatality or mortality, length of hospital stay, or readmissions. This indicates that healthcare of the patients in greatest need was not delayed in Norway, and that the observed decrease in admissions is mainly due to non-urgent disease. In addition, this study highlights the association between well-known infection control measures, such as handwashing and work absence for symptomatic individuals, and the burden of infections in general. The long-term effects of more intrusive infection control measures, such as isolation, and from fewer patients in total being admitted to hospital, remains to be seen.

## Supplemental Material

sj-docx-1-sjp-10.1177_14034948221082959 – Supplemental material for Emergency hospital admissions, prognosis, and population mortality in Norway during the first wave of the Covid-19 epidemic Click here for additional data file. Supplemental material, sj-docx-1-sjp-10.1177_14034948221082959 for Emergency hospital admissions, prognosis, and population mortality in Norway during the first wave of the Covid-19 epidemic by Henriette C. Jodal, Frederik E. Juul, Ishita Barua, Michael Bretthauer, Mette Kalager, Magnus Løberg and Louise Emilsson in Scandinavian Journal of Public Health
