# Supplementary material for: Emergency hospital admissions, prognosis, and population mortality in Norway during the first wave of the Covid-19 epidemic
Source: Scand J Public Health. 2022 May 12;50(6):795–802. doi: 10.1177/14034948221082959 (PMC9361465; doi:10.1177/14034948221082959)
Supplement: sj-docx-1-sjp-10.1177_14034948221082959 – Supplemental material for Emergency hospital admissions, prognosis, and population mortality in Norway during the first wave of the Covid-19 epidemic [file sj-docx-1-sjp-10.1177_14034948221082959.docx]

**Supplementary Appendix**

**Emergency Hospital Admissions, Prognosis and Population Mortality in Norway During the First Wave of the Covid-19 Epidemic**

**Table of content**

1. References
2. Figures and tables
   1. Figure S1: Weekly number of admissions among diagnoses probably unaffected by lockdown.
   2. Figure S2: Weekly number of admissions among diagnoses probably affected by lockdown.
   3. Table S1: Covid-19 mitigation measures during the first wave of the epidemic in Norway.
   4. Table S2: Number of admissions
   5. Table S3: The weekly number of all emergency admissions, admissions in the group probably unaffected by lockdown, and the group probably affected by lockdown.
   6. Table S4: Number of admissions in the diagnosis categories.
   7. Table S5: Number of admissions in the diagnosis categories, stratified by sex.
   8. Table S6: Number of admissions in the diagnosis categories, stratified by age group.
   9. Table S7: Number of deaths in hospital in the diagnosis categories.
   10. Table S8: Number of days in hospital in the diagnosis categories.
   11. Table S9: Number of weekly readmissions in the diagnosis categories.

**References**

1. Timeline: News from Norwegian Ministries about the Coronavirus disease Covid-19. 2020. <https://www.regjeringen.no/en/topics/koronavirus-covid-19/timeline-for-news-from-norwegian-ministries-about-the-coronavirus-disease-covid-19/id2692402/> (accessed 22nd February 2021).

2. Omfattende tiltak for å bekjempe koronaviruset. 2020. <https://www.regjeringen.no/no/aktuelt/nye-tiltak/id2693327/>.

3. Koronavirus sars-CoV-2 (coronavirus) – fakta, råd og tiltak. 2020. <https://www.fhi.no/nettpub/coronavirus/2020-11-29>).

4. Forskrift om smitteverntiltak mv. ved koronautbruddet (covid-19-forskriften). 2020. <https://lovdata.no/dokument/SF/forskrift/2020-03-27-470>

5. Vil åpne samfunnet gradvis og kontrollert. 2020. <https://www.regjeringen.no/no/aktuelt/Vil-apne-samfunnet-gradvis-og-kontrollert/id2697060/>.

6. Skolene åpner for alle elever fra 11. mai. 2020. <https://www.regjeringen.no/no/aktuelt/skolene-apner-for-alle-elever-fra-11.-mai/id2701512/>.

7. Nå åpner universitetene, høyskolene og fagskolene igjen. 2020. <https://www.regjeringen.no/no/aktuelt/apning-av-hoyskoler-universitet-og-fagskoler/id2706353/>.

***Figure S1:* Weekly number of admissions among diagnoses probably unaffected by lockdown: (A) acute myocardial infarction, (B) acute abdominal conditions and (C) cerebrovascular disease, years 2017-2020. The grey area marks the implementation of the Covid-19 mitigation measures.**

**
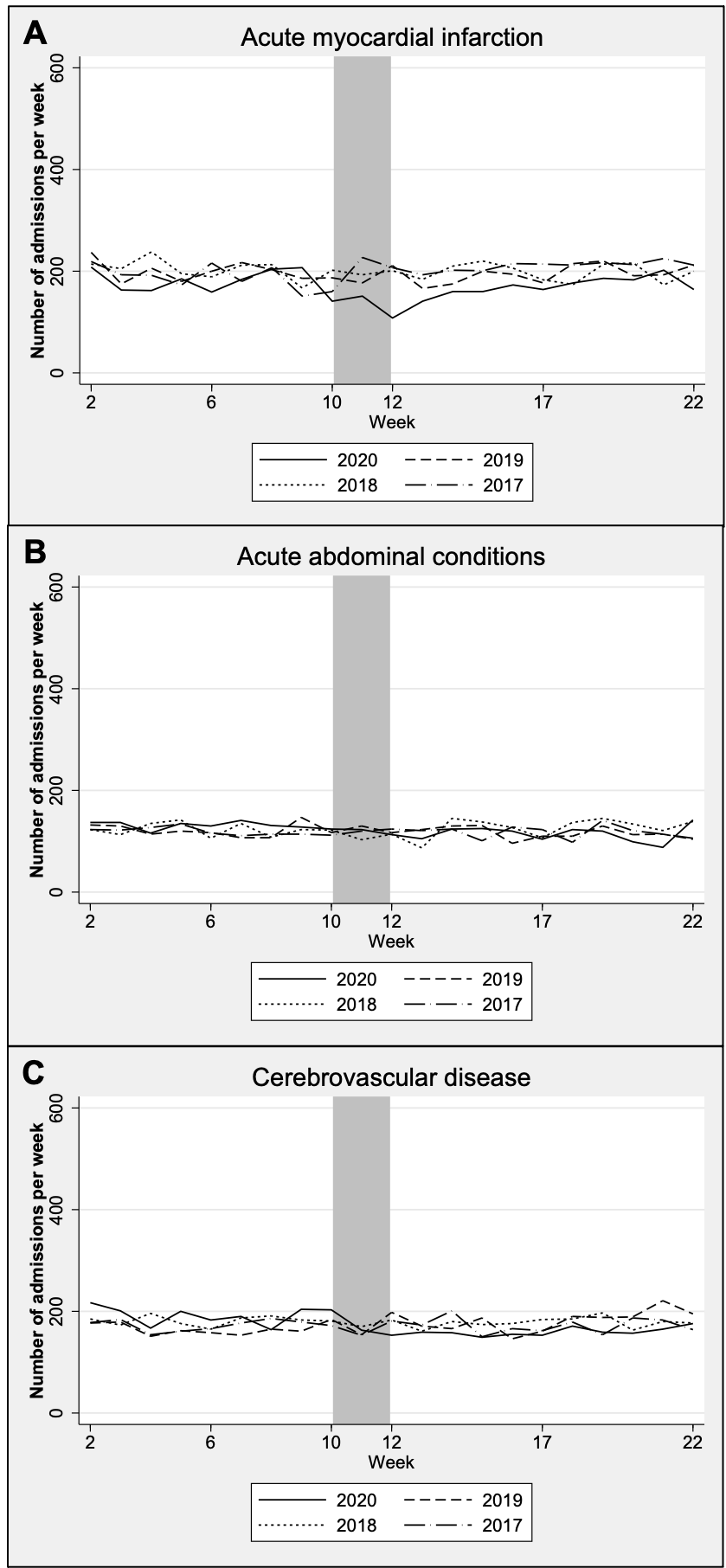
**

***Figure S2:* Weekly number of admissions among diagnoses probably affected by lockdown: (A) infections and (B) injuries, years 2017-2020. The grey area marks the implementation of the Covid-19 mitigation measures.**

***Table S1:* Covid-19 mitigation measures during the first wave of the epidemic in Norway**

| **MEASURE** | **WHEN** |
| --- | --- |
| **Daily life** |  |
| Travel restrictions | 12^th^ March, ongoing^1,2^ |
| Stay-at-home order | 12^th^ March to 20^th^ April^1-3^ |
| Sports and cultural events | Ban 12^th^ March-7^th^ May; <50 persons 7^th^ May; ^1,2^ <200 persons 15^th^ June^4^ |
| **Education** |  |
| Day care | Closed from 12^th^ March. ^1,2^ Gradual opening 27^th^ April.^5^ |
| Schools | Closed 12^th^ March. ^1,2^ Gradual opening 11^th^ May.^6^ |
| Universities and colleges | Closed 12^th^ March. ^1,2^ Online teaching, gradual opening 27^th^ April^37^ and full reopening 15^th^ June^7^ |
| **Health care** |  |
| Activity | Non-essential health care cancelled 12^th^ March-20^th^ April^3^ |

***Table S2:* Number of admissions.** The weekly number of all emergency admissions, admissions in the diagnosis group probably unaffected by lockdown, and the diagnosis group probably affected by lockdown, during the first wave of Covid-19 in 2020 (week 12-22), compared to mean of the same period previous years (2017, 2018 and 2019).

|  |  | **2020** | **Mean 2017-19** |
| --- | --- | --- | --- |
|  |  | **Mean** | **Mean** |
|  |  | **(lowest-highest)** | **(lowest-highest)** |
|  | **All emergency admissions** | 5477 | 6661 |
|  |  | (4616-6202) | (6144-7058) |
| **Unaffected by lockdown** | **Acute myocardial infarction** | 165 | 201 |
|  |  | (108-202) | (166-225) |
|  | **Abdominal conditions** | 108 | 110 |
|  |  | (83-134) | (80-137) |
|  | **Cerebrovascular disease** | 160 | 178 |
|  |  | (149-176) | (146-221) |
| **Affected by lockdown** | **Infections** | 141 | 274 |
|  |  | (98-133) | (208-383) |
|  | **Injuries** | 391 | 484 |
|  |  | (280-476) | (415-638) |

***Table S3:* The weekly number of all emergency admissions, admissions in the group probably unaffected by lockdown, and the group probably affected by lockdown. Absolute difference (AD) and relative risk (RR) is calculated with the mean of years 2017-19 as the reference, separately for the weekly period preceding the first wave (week 2-10) and the during first wave of Covid-19 (week 12-22).**

|  |  | **Week 2-10** | | | | | | **Week 12-22** | | | | | |
| --- | --- | --- | --- | --- | --- | --- | --- | --- | --- | --- | --- | --- | --- |
| **Diagnosis group** | **Year** | **No. of admissions** | **Lowest-highest** | **AD** | **CI (95%)** | **RR** | **CI (95%)** | **No. of admissions** | **Lowest-highest** | **AD** | **CI (95%)** | **RR** | **CI (95%)** |
| All emergency admissions | Mean 2017-19 | 6810 | (6392-7081) | 0 |  | 1.00 |  | 6661 | (6144-7058) | 0 |  | 1.00 |  |
|  | 2020 | 6650 | (6443-6873) | -160 | (-221 - -98) | 0.98 | (0.97-0.99) | 5477 | (4616-6202) | -1184 | (-1236) | 0.82 | (0.81-0.83) |
|  | 2019 | 6795 | (6623-6901) | -14 | (-76 - 48) | 1.00 | (0.99-1.01) | 6615 | (6417-6982) | -46 | (-101 - 10) | 0.99 | (0.98-1.00) |
|  | 2018 | 6898 | (6675-7081) | 89 | (26 - 151) | 1.01 | (1.00-1.02) | 6793 | (6449-7058) | 133 | (77-189) | 1.02 | (1.01-1.03) |
|  | 2017 | 6735 | (6392-6977) | -75 | (-137 - -13) | 0.99 | (0.98-1.00) | 6574 | (6144-6746) | -87 | (-142 - -32) | 0.99 | (0.98-1.00) |
| Unaffected by lockdown | Mean 2017-19 | 480 | (427-550) | 0 |  | 1.00 |  | 489 | (424-542) | 0 |  | 1.00 |  |
|  | 2020 | 496 | (441-553) | 15 | (-2 - 32) | 1.03 | (1.00-1.07) | 433 | (369-474) | -56 | (-71 - - 42) | 0.88 | (0.86-0.91) |
|  | 2019 | 477 | (452-540) | -3 | (-20 - 13) | 0.99 | (0.96-1.03) | 486 | (424-525) | -3 | (-18 - 12) | 0.99 | (0.96-1.03) |
|  | 2018 | 499 | (459-550) | 18 | (2 - 35) | 1.04 | (1.00-1.07) | 492 | (425-542) | 3 | (-12 - 18) | 1.01 | (0.97-1.04) |
|  | 2017 | 466 | (427-505) | -15 | (-31 - 1) | 0.97 | (0.94-1.00) | 490 | (442-520) | 0 | (-15 - 15) | 1.00 | (0.97-1.03) |
| Affected by lockdown | Mean 2017-19 | 941 | (818-1121) | 0 |  | 1.00 |  | 759 | (665-976) | 0 |  | 1.00 |  |
|  | 2020 | 801 | (750-855) | -139 | (-161 - -118) | 0.85 | (0.83-0.87) | 532 | (465-597) | -227 | (-243 - -211) | 0.70 | (0.68-0.72) |
|  | 2019 | 890 | (818-1067) | -50 | (-73 - -28) | 0.95 | (0.92-0.97) | 747 | (689-848) | -12 | (-30 - 7) | 0.98 | (0.96-1.01) |
|  | 2018 | 1033 | (947-1121) | 92 | (68 - 116) | 1.10 | (1.07-1.12) | 787 | (665-976) | 28 | (9 - 47) | 1.04 | (1.01-1.06) |
|  | 2017 | 899 | (822-961) | -42 | (-64 - -19) | 0.96 | (0.93-0.98) | 743 | (712-773) | -16 | (-35 - 2) | 0.98 | (0.95-1.00) |

***Table S4:* Number of admissions in the diagnosis categories. Absolute difference (AD) and relative risk (RR) is calculated with the mean of years 2017-19 as the reference, separately for the weekly period preceding the first wave (week 2-10) and the during first wave of Covid-19 (week 12-22).**

|  |  |  | **Week 2-10** | | | | **Week 12-22** | | | |
| --- | --- | --- | --- | --- | --- | --- | --- | --- | --- | --- |
| **Diagnosis group** | **Diagnosis category** | **Year** | **AD** | **CI (95%)** | **RR** | **CI (95%)** | **AD** | **CI (95%)** | **RR** | **CI (95%)** |
| Unaffected by lockdown | Acute myocardial infarction | Mean 2017-19 | 0 |  | 1.00 |  | 0 |  | 1.00 |  |
|  |  | 2020 | -18 | (-28- -7) | 0.91 | (0.86-0.96) | -36 | (-45 - -27) | 0.82 | (0.78-0.86) |
|  |  | 2019 | 2 | (-9 - 13) | 1.01 | (0.96-1.07) | -6 | (-15 - 4) | 0.97 | (0.93-1.02) |
|  |  | 2018 | 7 | (-4 - 18) | 1.04 | (0.98-1.09) | -3 | (-13 - 7) | 0.98 | (0.94-1.03) |
|  |  | 2017 | -9 | (-19 - 1) | 0.95 | (0.90-1.01) | 9 | (-1 - 19) | 1.04 | (0.99-1.09) |
|  | Acute abdominal conditions | Mean 2017-19 | 0 |  | 1.00 |  | 0 |  | 1.00 |  |
|  |  | 2020 | 14 | (6 - 22) | 1.13 | (1.05-1.21) | -2 | (-9 - 5) | 0.98 | (0.92-1.05) |
|  |  | 2019 | 2 | (-5 - 10) | 1.02 | (0.95-1.10) | -2 | (-9 - 5) | 0.98 | (0.92-1.04) |
|  |  | 2018 | 3 | (-5 - 11) | 1.03 | (0.96-1.10) | 6 | (-2 - 13) | 1.05 | (0.98-1.12) |
|  |  | 2017 | -5 | (-13 - 2) | 0.95 | (0.88-1.02) | -3 | (-10 - 4) | 0.97 | (0.91-1.04) |
|  | Cerebrovascular disease | Mean 2017-19 | 0 |  | 1.00 |  | 0 |  | 1.00 |  |
|  |  | 2020 | 19 | (8 - 29) | 1.11 | (1.05-1.17) | -18 | (-27 - 10) | 0.90 | (0.85-0.95) |
|  |  | 2019 | -8 | (-18 - 2) | 0.95 | (0.90-1.01) | 5 | (-4 - 14) | 1.03 | (0.98-1.08) |
|  |  | 2018 | 8 | (-2 - 19) | 1.05 | (0.99-1.11) | 0 | (-9 - 9) | 1.00 | (0.95-1.05) |
|  |  | 2017 | 0 | (-10 - 9) | 1.00 | (0.94-1.06) | -5 | (-14 - 4) | 0.97 | (0.92-1.02) |
| Affected by lockdown | Infections | Mean 2017-19 | 0 |  | 1.00 |  | 0 |  | 1.00 |  |
|  |  | 2020 | -88 | (-102 - -74) | 0.78 | (0.75-0.82) | -133 | (-143 - -125) | 0.51 | (0.49-0.54) |
|  |  | 2019 | -23 | (-38 - -8) | 0.94 | (0.91-0.98) | 6 | (-6 - 17) | 1.02 | (0.98-1.06) |
|  |  | 2018 | 86 | (70 - 102) | 1.21 | (1.17-1.26) | 0 | (-12 - 11) | 1.00 | (0.96-1.04) |
|  |  | 2017 | -63 | (-77 - -48) | 0.85 | (0.81-0.88) | -5 | (-17 - 6) | 0.98 | (0.94-1.02) |
|  | Injuries | Mean 2017-19 | 0 |  | 1.00 |  | 0 |  | 1.00 |  |
|  |  | 2020 | -52 | (-69 - -35) | 0.90 | (0.87-0.93) | -94 | (-107 - -80) | 0.81 | (0.78-0.83) |
|  |  | 2019 | -27 | (-44 - -10) | 0.95 | (0.92-0.98) | -18 | (-32 - -3) | 0.96 | (0.93-0.99) |
|  |  | 2018 | 6 | (-12 - 23) | 1.01 | (0.98-1.04) | 29 | (13 - 44) | 1.06 | (1.03-1.09) |
|  |  | 2017 | 21 | (4 - 39) | 1.04 | (1.01-1.07) | -11 | (-26 - 4) | 0.98 | (0.95-1.01) |

***Table S5:* Number of admissions in the diagnosis categories, stratified by sex. Absolute difference (AD) and relative risk (RR) is calculated with the mean of years 2017-19 as the reference, separately for the weekly period preceding the first wave (week 2-10) and the during first wave of Covid-19 (week 12-22).**

|  |  |  |  | **Week 2-10** | | | | | | **Week 12-22** | | | | | |
| --- | --- | --- | --- | --- | --- | --- | --- | --- | --- | --- | --- | --- | --- | --- | --- |
| **Diagnosis group** | **Diagnosis category** | **Sex** | **Year** | **No. of admissions** | **Lowest-highest** | **AD** | **CI (95%)** | **RR** | **CI (95%)** | **No. of admissions** | **Lowest-highest** | **AD** | **CI (95%)** | **RR** | **CI (95%)** |
| Unaffected by lockdown | Acute myocardial infarction | Women | Mean 2017-19 | 58 | (43-74) | 0 |  | 1.00 |  | 59 | (37-84) | 0 |  | 1.00 |  |
|  |  |  | 2020 | 50 | (37-63) | -8 | (-14 - - 3) | 0.86 | (0.77-0.96) | 49 | (34-74) | -9 | (-14 - 4) | 0.84 | (0.76-0.93) |
|  |  |  | 2019 | 63 | (46-74) | 5 | (-1 - 11) | 1.08 | (0.98-1.19) | 54 | (37-70) | -5 | (-10 - 0) | 0.92 | (0.84-1.01) |
|  |  |  | 2018 | 56 | (44-64) | -2 | (-8 - 3) | 0.96 | (0.86-1.06) | 59 | (41-84) | 0 | (-5 - 6) | 1.01 | (0.92-1.10) |
|  |  |  | 2017 | 56 | (43-61) | -2 | (-8 - 3) | 0.96 | (0.87-1.06) | 63 | (49-76) | 4 | (-1 - 10) | 1.08 | (0.99-1.17) |
|  |  | Men | Mean 2017-19 | 139 | (90-182) | 0 |  | 1.00 |  | 143 | (109-165) | 0 |  | 1.00 |  |
|  |  |  | 2020 | 129 | (100-159) | -10 | (-18 - -1) | 0.93 | (0.87-0.99) | 116 | (74-143) | -27 | (-34 - -19) | 0.81 | (0.76-0.86) |
|  |  |  | 2019 | 126 | (118-167) | -3 | (-12 - 6) | 0.98 | (0.92-1.05) | 142 | (109-159) | -1 | (-9 - 7) | 0.99 | (0.94-1.05) |
|  |  |  | 2018 | 148 | (108-182) | 9 | (0 - 19) | 1.07 | (1.00-1.14) | 139 | (112-165) | -3 | (-12 - 5) | 0.98 | (0.92-1.03) |
|  |  |  | 2017 | 132 | (90-165) | -7 | (-15 - 2) | 0.95 | (0.89-1.02) | 147 | (135-164) | 4 | (-4 - 12) | 1.03 | (0.97-1.09) |
|  | Acute abdominal conditions | Women | Mean 2017-19 | 54 | (40-65) | 0 |  | 1.00 |  | 55 | (35-71) | 0 |  | 1.00 |  |
|  |  |  | 2020 | 61 | (46-68) | 7 | (1 - 12) | 1.12 | (1.02-1.24) | 53 | (42-65) | -2 | (-7 - 3) | 0.97 | (0.88-1.06) |
|  |  |  | 2019 | 54 | (41-60) | 0 | (-6 - 5) | 1.00 | (0.90-1.11) | 54 | (45-63) | 0 | (-5 - 5) | 1.00 | (0.91-1.09) |
|  |  |  | 2018 | 55 | (40-65) | 1 | (-5 - 6) | 1.01 | (0.91-1.12) | 56 | (35-71) | 1 | (-4 - 7) | 1.03 | (0.93-1.13) |
|  |  |  | 2017 | 53 | (44-61) | -1 | (-6 - 5) | 0.99 | (0.89-1.10) | 53 | (41-66) | -1 | (-6 - 4) | 0.98 | (0.89-1.07) |
|  |  | Men | Mean 2017-19 | 56 | (43-78) | 0 |  | 1.00 |  | 55 | (37-75) | 0 |  | 1.00 |  |
|  |  |  | 2020 | 64 | (52-76) | 7 | (2 - 13) | 1.13 | (1.03-1.25) | 55 | (41-70) | 0 | (-5 - 5) | 1.00 | (0.91-1.09) |
|  |  |  | 2019 | 59 | (44-74) | 3 | (-3 - 8) | 1.05 | (0.95-1.16) | 53 | (39-66) | -2 | (-7 - 3) | 0.96 | (0.87-1.05) |
|  |  |  | 2018 | 58 | (47-78) | 2 | (-4 - 8) | 1.04 | (0.94-1.15) | 60 | (45-75) | 4 | (-1 - 9) | 1.07 | (0.98-1.17) |
|  |  |  | 2017 | 51 | (43-64) | -5 | (-10 - 1) | 0.92 | (0.82-1.02) | 54 | (37-75) | -2 | (-7 - 3) | 0.97 | (0.88-1.06) |
|  | Cerebrovascular disease | Women | Mean 2017-19 | 81 | (67-95) | 0 |  | 1.00 |  | 79 | (54-100) | 0 |  | 1.00 |  |
|  |  |  | 2020 | 89 | (76-105) | 8 | (1 - 15) | 1.10 | (1.01-1.20) | 70 | (61-78) | -10 | (-15 - -4) | 0.88 | (0.81-0.95) |
|  |  |  | 2019 | 79 | (72-95) | -2 | (-8 - 5) | 0.98 | (0.90-1.07) | 84 | (67-100) | 4 | (-2 - 10) | 1.05 | (0.98-1.14) |
|  |  |  | 2018 | 83 | (67-90) | 2 | (-5 - 9) | 1.03 | (0.95-1.12) | 78 | (59-89) | -1 | (-7 - 5) | 0.99 | (0.91-1.07) |
|  |  |  | 2017 | 80 | (68-94) | -1 | (-7 - 6) | 0.99 | (0.91-1.08) | 76 | (54-86) | -3 | (-9 - 3) | 0.96 | (0.88-1.04) |
|  |  | Men | Mean 2017-19 | 93 | (74-114) | 0 |  | 1.00 |  | 99 | (65-125) | 0 |  | 1.00 |  |
|  |  |  | 2020 | 103 | (87-112) | 10 | (3 - 18) | 1.11 | (1.03-1.20) | 90 | (79-102) | -9 | (-15 - - 2) | 0.91 | (0.85-0.98) |
|  |  |  | 2019 | 86 | (74-108) | -6 | (-13 - 1) | 0.93 | (0.86-1.01) | 99 | (67-125) | 1 | (-6 - 8) | 1.01 | (0.94-1.08) |
|  |  |  | 2018 | 99 | (84-114) | 6 | (-1 - 14) | 1.07 | (0.99-1.15) | 100 | (86-114) | 1 | (-6 - 8) | 1.01 | (0.94-1.08) |
|  |  |  | 2017 | 93 | (79-107) | 0 | (-7 - 7) | 1.00 | (0.93-1.08) | 97 | (65-116) | -2 | (-9 - 5) | 0.98 | (0.92-1.05) |
| Affected by lockdown | Infections | Women | Mean 2017-19 | 194 | (124-264) | 0 |  | 1.00 |  | 127 | (97-173) | 0 |  | 1.00 |  |
|  |  |  | 2020 | 155 | (132-200) | -40 | (-49 - -30) | 0.80 | (0.75-0.84) | 56 | (26-87) | -71 | (-77 - -65) | 0.44 | (0.40-0.48) |
|  |  |  | 2019 | 179 | (124-223) | -15 | (-26 - -5) | 0.92 | (0.87-0.97) | 121 | (108-152) | 3 | (-5 - 11) | 1.03 | (0.97-1.09) |
|  |  |  | 2018 | 239 | (212-264) | 44 | (33 - 56) | 1.23 | (1.17-1.29) | 127 | (97-173) | -1 | (-8 - 7) | 1.00 | (0.94-1.06) |
|  |  |  | 2017 | 165 | (143-195) | -29 | (-49 - -19) | 0.85 | (0.80-0.90) | 125 | (108-140) | -3 | (-10 - 5) | 0.98 | (0.92-1.04) |
|  |  | Men | Mean 2017-19 | 210 | (151-303) | 0 |  | 1.00 |  | 147 | (111-210) | 0 |  | 1.00 |  |
|  |  |  | 2020 | 162 | (143-194) | -48 | (-58 - -38) | 0.77 | (0.73-0.82) | 85 | (62-106) | -62 | (-69 - -55) | 0.58 | (0.54-0.62) |
|  |  |  | 2019 | 202 | (160-247) | -8 | (-19 - 3) | 0.96 | (0.91-1.01) | 150 | (118-182) | 3 | (-6 - 11) | 1.01 | (0.96-1.08) |
|  |  |  | 2018 | 252 | (208-303) | 42 | (30 - 53) | 1.20 | (1.14-1.26) | 147 | (111-210) | 0 | (-8 - 8) | 1.00 | (0.94-1.06) |
|  |  |  | 2017 | 176 | (151-216) | -34 | (-44 - -23) | 0.84 | (0.79-0.89) | 145 | (122-173) | -3 | (-11 - 6) | 0.98 | (0.93-1.04) |
|  | Injuries | Women | Mean 2017-19 | 283 | (228-351) | 0 |  | 1.00 |  | 244 | (193-338) | 0 |  | 1.00 |  |
|  |  |  | 2020 | 246 | (223-266) | -37 | (-49 - -25) | 0.87 | (0.83-0.91) | 206 | (163-237) | -38 | (-48 - -27) | 0.85 | (0.81-0.89) |
|  |  |  | 2019 | 269 | (228-303) | -14 | (-27 - -2) | 0.95 | (0.91-0.99) | 236 | (212-272) | -8 | (-19 - 2) | 0.97 | (0.92-1.01) |
|  |  |  | 2018 | 295 | (252-351) | 12 | (-1 - 25) | 1.04 | (1.00-1.09) | 262 | (193-338) | 18 | (7 - 29) | 1.07 | (1.03-1.12) |
|  |  |  | 2017 | 285 | (252-331) | 2 | (-10 - 15) | 1.01 | (0.96-1.05) | 234 | (210-266) | -10 | (-20 - 1) | 0.96 | (0.92-1.00) |
|  |  | Men | Mean 2017-19 | 253 | (222-304) | 0 |  | 1.00 |  | 240 | (202-300) | 0 |  | 1.00 |  |
|  |  |  | 2020 | 238 | (195-266) | -15 | (-27 - -3) | 0.94 | (0.90-0.99) | 184 | (117-260) | -56 | (-65 - -46) | 0.77 | (0.73-0.81) |
|  |  |  | 2019 | 240 | (222-294) | -13 | (-25 - -1) | 0.95 | (0.90-1.00) | 231 | (208-257) | -9 | (-20 - 1) | 0.96 | (0.92-1.01) |
|  |  |  | 2018 | 247 | (222-297) | -6 | (-18 - 6) | 0.98 | (0.93-1.02) | 251 | (204-300) | 10 | (0 - 21) | 1.04 | (1.00-1.09) |
|  |  |  | 2017 | 272 | (229-304) | 19 | (7 - 31) | 1.07 | (1.03-1.13) | 239 | (202-281) | -1 | (-12 - 9) | 1.00 | (0.96-1.04) |

***Table S6:* Number of admissions in the diagnosis categories, stratified by age group: 0-44 years, 45-64 years, and 65 years and older. Absolute difference (AD) and relative risk (RR) is calculated with the mean of years 2017-19 as the reference, separately for the weekly period preceding the first wave (week 2-10) and the during first wave of Covid-19 (week 12-22).**

|  |  |  |  | **Week 2-10** | | | | | | **Week 12-22** | | | | | |
| --- | --- | --- | --- | --- | --- | --- | --- | --- | --- | --- | --- | --- | --- | --- | --- |
| **Diagnosis group** | **Diagnosis category** | **Age** | **Year** | **No. of admissions** | **Lowest-highest** | **AD** | **CI (95%)** | **RR** | **CI (95%)** | **No. of admissions** | **Lowest-highest** | **AD** | **CI (95%)** | **RR** | **CI (95%)** |
| Unaffected by lockdown | Acute myocardial infarction | 0-44 years | Mean 2017-19 | 8 | (1-16) | 0 |  | 1.00 |  | 6 | (1-12) | 0 |  | 1.00 |  |
|  |  |  | 2020 | 6 | (2-13) | -2 | (-4 - 0) | 0.77 | (0.56-1.05) | 5 | (2-10) | -1 | (-2 - 1) | 0.89 | (0.65-1.20) |
|  |  | 45-64 years | Mean 2017-19 | 66 | (35-91) | 0 |  | 1.00 |  | 69 | (33-87) | 0 |  | 1.00 |  |
|  |  |  | 2020 | 59 | (38-84) | -7 | (-13 - -1) | 0.89 | (0.81-0.98) | 55 | (37-66) | -14 | (-19 - -8) | 0.80 | (0.73-0.88) |
|  |  | ≥65 years | Mean 2017-19 | 109 | (67-154) | 0 |  | 1.00 |  | 109 | (66-148) | 0 |  | 1.00 |  |
|  |  |  | 2020 | 97 | (77-117) | -12 | (-19 - -4) | 0.89 | (0.83-0.96) | 83 | (27-107) | -26 | (-33 - -20) | 0.76 | (0.71-0.82) |
|  | Acute abdominal conditions | 0-44 years | Mean 2017-19 | 54 | (42-73) | 0 |  | 1.00 |  | 52 | (31-81) | 0 |  | 1.00 |  |
|  |  |  | 2020 | 57 | (45-66) | 3 | (-3 - 9) | 1.05 | (0.95-1.16) | 51 | (39-63) | -1 | (-6 - 4) | 0.99 | (0.89-1.09) |
|  |  | 45-64 years | Mean 2017-19 | 14 | (2-28) | 0 |  | 1.00 |  | 15 | (2-28) | 0 |  | 1.00 |  |
|  |  |  | 2020 | 13 | (5-21) | -1 | (-4 - 2) | 0.92 | (0.74-1.13) | 14 | (5-24) | -1 | (-3 - 2) | 0.96 | (0.79-1.15) |
|  |  | ≥65 years | Mean 2017-19 | 9 | (0-22) | 0 |  | 1.00 |  | 9 | (0-23) | 0 |  | 1.00 |  |
|  |  |  | 2020 | 14 | (5-25) | 5 | (2 - 8) | 1.55 | (1.24-1.92) | 7 | (0-11) | -2 | (-4 - 0) | 0.79 | (0.61-1.02) |
|  | Cerebrovascular disease | 0-44 years | Mean 2017-19 | 7 | (2-18) | 0 |  | 1.00 |  | 8 | (2-16) | 0 |  | 1.00 |  |
|  |  |  | 2020 | 7 | (5-11) | 0 | (-2 - 2) | 1.06 | (0.79-1.41) | 6 | (2-11) | -2 | (-3 - 0) | 0.77 | (0.58-1.02) |
|  |  | 45-64 years | Mean 2017-19 | 27 | (13-40) | 0 |  | 1.00 |  | 27 | (12-43) | 0 |  | 1.00 |  |
|  |  |  | 2020 | 30 | (19-47) | 4 | (0 - 8) | 1.14 | (1.00-1.32) | 23 | (11-34) | -4 | (-7 - -1) | 0.85 | (0.74-0.98) |
|  |  | ≥65 years | Mean 2017-19 | 104 | (68-129) | 0 |  | 1.00 |  | 108 | (81-143) | 0 |  | 1.00 |  |
|  |  |  | 2020 | 121 | (96-140) | 18 | (10 - 26) | 1.17 | (1.09-1.26) | 95 | (85-106) | -12 | (-19 - -6) | 0.88 | (0.83-0.95) |
| Affected by lockdown | Infections | 0-44 years | Mean 2017-19 | 55 | (34-75) | 0 |  | 1.00 |  | 206 | (165-259) | 0 |  | 1.00 |  |
|  |  |  | 2020 | 53 | (39-70) | -2 | (-7 - 4) | 0.97 | (0.87-1.07) | 194 | (161-220) | -20 | (-23 - -17) | 0.38 | (0.31-0.45) |
|  |  | 45-64 years | Mean 2017-19 | 56 | (32-82) | 0 |  | 1.00 |  | 33 | (8-49) | 0 |  | 1.00 |  |
|  |  |  | 2020 | 37 | (27-46) | -20 | (-24 - -15) | 0.65 | (0.58-0.74) | 12 | (6-21) | -17 | (-20 - -14) | 0.45 | (0.38-0.53) |
|  |  | ≥65 years | Mean 2017-19 | 252 | (158-370) | 0 |  | 1.00 |  | 31 | (8-51) | 0 |  | 1.00 |  |
|  |  |  | 2020 | 177 | (154-223) | -75 | (-85 - -65) | 0.70 | (0.66-0.74) | 14 | (4-23) | -85 | (-93 - -79) | 0.49 | (0.46-0.53) |
|  | Injuries | 0-44 years | Mean 2017-19 | 159 | (121-210) | 0 |  | 1.00 |  | 165 | (132-243) | 0 |  | 1.00 |  |
|  |  |  | 2020 | 142 | (117-167) | -17 | (-26 - -8) | 0.89 | (0.84-0.95) | 111 | (132-243) | -54 | (-62 - -47) | 0.67 | (0.63-0.71) |
|  |  | 45-64 years | Mean 2017-19 | 117 | (84-165) | 0 |  | 1.00 |  | 91 | (61-159) | 0 |  | 1.00 |  |
|  |  |  | 2020 | 98 | (78-111) | -18 | (-26 - -11) | 0.84 | (0.78-0.91) | 70 | (39-88) | -21 | (-27 - -15) | 0.77 | (0.71-0.83) |
|  |  | ≥65 years | Mean 2017-19 | 242 | (201-293) | 0 |  | 1.00 |  | 206 | (165-259) | 0 |  | 1.00 |  |
|  |  |  | 2020 | 225 | (179-261) | -18 | (-29 - -6) | 0.93 | (0.88-0.97) | 194 | (161-220) | -12 | (-22 - -3) | 0.94 | (0.89-0.99) |

***Table S7:* Number of deaths in hospital in the diagnosis categories. Absolute difference (AD) and in-hospital fatality (RR) is calculated with the mean of years 2017-19 as the reference, separately for the weekly period preceding the first wave (week 2-10) and the during first wave of Covid-19 (week 12-22).**

|  |  |  | **Week 2-10** | | | | | | **Week 12-22** | | | | | |
| --- | --- | --- | --- | --- | --- | --- | --- | --- | --- | --- | --- | --- | --- | --- |
| **Diagnosis group** | **Diagnosis category** | **Year** | **No. of deaths** | **Lowest-highest** | **AD** | **Lowest-highest** | **RR** | **CI (95%)** | **No. of deaths** | **Lowest-highest** | **AD** | **Lowest-highest** | **RR** | **CI (95%)** |
| Unaffected by lockdown | Acute myocardial infarction | Mean 2017-19 | 5 | (1-12) | 0 |  | 1.00 |  | 5 | (1-8) | 0 |  | 1.00 |  |
|  |  | 2020 | 3 | (0-5) | -3 | (-4 - -1) | 0.51 | (0.31-0.79) | 3 | (0-6) | -2 | (-3 - 0) | 0.66 | (0.44-0.96) |
|  |  | 2019 | 5 | (1-7) | 0 | (-2 - 1) | 0.93 | (0.65-1.31) | 4 | (1-6) | -1 | (-2 - 0) | 0.77 | (0.53-1.11) |
|  |  | 2018 | 4 | (2-6) | -1 | (-3 - 0) | 0.74 | (0.50-1.08) | 5 | (3-7) | 1 | (-1 - 2) | 1.11 | (0.80-1.52) |
|  |  | 2017 | 7 | (1-12) | 2 | (0 - 4) | 1.33 | (0.97-1.80) | 5 | (2-8) | 1 | (-1 - 2) | 1.11 | (0.80-1.52) |
|  | Acute abdominal conditions | Mean 2017-19 | 1 | (0-5) | 0 |  | 1.00 |  | 2 | (0-6) | 0 |  | 1.00 |  |
|  |  | 2020 | 2 | (1-4) | 0 | (-1 - 1) | 1.15 | (0.59-2.14) | 1 | (0-2) | -1 | (-1 - 0) | 0.52 | (0.22-1.06) |
|  |  | 2019 | 1 | (0-4) | 0 | (-1 - 1) | 0.92 | (0.44-1.80) | 1 | (0-6) | 0 | (-1 - 1) | 0.92 | (0.49-1.64) |
|  |  | 2018 | 2 | (0-4) | 0 | (-1 - 1) | 1.23 | (0.64-2.25) | 2 | (0-4) | 0 | (-1 - 1) | 0.98 | (0.53-1.72) |
|  |  | 2017 | 1 | (0-5) | 0 | (-1 - 1) | 0.85 | (0.39-1.68) | 2 | (0-4) | 0 | (-1 - 1) | 1.10 | (0.61-1.89) |
|  | Cerebrovascular disease | Mean 2017-19 | 9 | (4-17) | 0 |  | 1.00 |  | 8 | (2-14) | 0 |  | 1.00 |  |
|  |  | 2020 | 9 | (3-16) | 0 | (-3 - 2) | 0.96 | (0.74-1.23) | 9 | (4-14) | 1 | (-1 - 4) | 1.19 | (0.94-1.50) |
|  |  | 2019 | 9 | (5-11) | -1 | (-3 - 1) | 0.91 | (0.70-1.18) | 9 | (5-14) | 1 | (-1 - 3) | 1.17 | (0.92-1.47) |
|  |  | 2018 | 10 | (7-13) | 0 | (-2 - 3) | 1.03 | (0.80-1.32) | 7 | (5-12) | -1 | (-3 - 1) | 0.89 | (0.68-1.15) |
|  |  | 2017 | 10 | (4-17) | 1 | (-2 - 3) | 1.06 | (0.82-1.35) | 7 | (2-13) | 0 | (-2 - 1) | 0.95 | (0.73-1.22) |
| Affected by lockdown | Infections | Mean 2017-19 | 22 | (10-33) | 0 |  | 1.00 |  | 15 | (5-30) | 0 |  | 1.00 |  |
|  |  | 2020 | 16 | (10-20) | -6 | (-9 - -2) | 0.73 | (0.60-0.87) | 12 | (5-19) | -3 | (-5 - 0) | 0.81 | (0.67-0.99) |
|  |  | 2019 | 19 | (10-22) | -3 | (-7 - 0) | 0.84 | (0.70-1.00) | 16 | (12-19) | 1 | (-2 - 3) | 1.04 | (0.87-1.24) |
|  |  | 2018 | 28 | (17-33) | 5 | (1 - 9) | 1.22 | (1.04-1.41) | 15 | (5-30) | 0 | (-3 - 2) | 0.98 | (0.81-1.17) |
|  |  | 2017 | 21 | (13-30) | -1 | (-5 - 2) | 0.94 | (0.80-1.11) | 15 | (7-23) | 0 | (-3 - 2) | 0.98 | (0.82-1.18) |
|  | Injuries | Mean 2017-19 | 6 | (2-11) | 0 |  | 1.00 |  | 5 | (1-9) | 0 |  | 1.00 |  |
|  |  | 2020 | 6 | (2-15) | 0 | (-2 - 2) | 0.99 | (0.71-1.37) | 4 | (2-8) | -1 | (-2 - 1) | 0.87 | (0.61-1.20) |
|  |  | 2019 | 4 | (2-7) | -1 | (-3 - 0) | 0.76 | (0.52-1.09) | 5 | (2-9) | 0 | (-1 - 2) | 1.09 | (0.79-1.47) |
|  |  | 2018 | 7 | (2-10) | 1 | (-1 - 3) | 1.17 | (0.85-1.58) | 4 | (1-9) | -1 | (-2 - 1) | 0.87 | (0.61-1.20) |
|  |  | 2017 | 6 | (4-11) | 0 | (-1 - 2) | 1.07 | (0.77-1.47) | 5 | (3-8) | 0 | (-1 - 2) | 1.05 | (0.76-1.43) |

***Table S8:* Number of days in hospital in the diagnosis categories. Absolute difference (AD) is calculated with the mean of years 2017-19 as the reference, separately for the weekly period preceding the first wave (week 2-10) and the during first wave of Covid-19 (week 12-22).**

|  |  |  | **Week 2-10** | | | | **Week 12-22** | | | |
| --- | --- | --- | --- | --- | --- | --- | --- | --- | --- | --- |
| **Diagnosis group** | **Diagnosis category** | **Year** | **No. of days in hospital** | **CI (95%)** | **AD** | **CI (95%)** | **No. of days in hospital** | **CI (95%)** | **AD** | **CI (95%)** |
| Unaffected by lockdown | Acute myocardial infarction | Mean 2017-19 | 2.63 | (2.51-2.74) | 0.00 |  | 2.62 | (2.51-2.73) | 0.00 |  |
|  |  | 2020 | 2.66 | (2.44-2.88) | 0.03 | (-0.20 - 0.27) | 2.48 | (2.32-2.64) | -0.15 | (-0.35 - 0.06) |
|  |  | 2019 | 2.45 | (2.24-2.65) | -0.18 | (-0.41 - 0.05) | 2.61 | (2.40-2.83) | -0.01 | (-0.23 - 0.22) |
|  |  | 2018 | 2.64 | (2.49-2.79) | 0.01 | (-0.21 - 0.22) | 2.49 | (2.37-2.60) | -0.13 | (-0.34 - 0.08) |
|  |  | 2017 | 2.80 | (2.57-3.03) | 0.17 | (-0.07 - 0.41) | 2.76 | (2.59-2.93) | 0.14 | (-0.07 - 0.35) |
|  | Abdominal conditions | Mean 2017-19 | 4.14 | (3.81-4.47) | 0.00 |  | 4.31 | (3.99-4.62) | 0.00 |  |
|  |  | 2020 | 3.94 | (3.54-4.34) | -0.20 | (-0.81 - 0.42) | 3.73 | (3.36-4.10) | -0.58 | (-1.17 - 0.01) |
|  |  | 2019 | 4.21 | (3.48-4.94) | 0.07 | (-0.64 - 0.78) | 4.40 | (3.91-4.88) | 0.09 | (-0.52 - 0.70) |
|  |  | 2018 | 3.89 | (3.47-4.30) | -0.25 | (-0.87 - 0.37) | 4.18 | (3.69-4.67) | -0.12 | (-0.74 - 0.49) |
|  |  | 2017 | 4.33 | (3.79-4.86) | 0.18 | (-0.47 - 0.84) | 4.34 | (3.68-5.00) | 0.03 | (-0.63 - 0.70) |
|  | Cerebrovascular disease | Mean 2017-19 | 4.51 | (4.24-4.80) | 0.00 |  | 4.54 | (4.28-4.80) | 0.00 |  |
|  |  | 2020 | 4.05 | (3.62-4.48) | -0.47 | (-1.01 - 0.07) | 3.87 | (3.49-4.25) | -0.67 | (-1.17 - -0.17) |
|  |  | 2019 | 4.55 | (4.09-5.01) | 0.03 | (-0.52 - 0.58) | 4.59 | (4.13-5.05) | 0.05 | (-0.48 - 0.57) |
|  |  | 2018 | 4.47 | (3.94-5.00) | -0.05 | (-0.62 - 0.52) | 4.39 | (3.96-4.82) | -0.16 | (-0.67 - 0.36) |
|  |  | 2017 | 4.53 | (4.06-5.01) | 0.02 | (-0.54 - 0.57) | 4.65 | (4.18-5.13) | 0.11 | (-0.42 - 0.64) |
| Affected by lockdown | Infections | Mean 2017-19 | 5.76 | (5.40-6.12) | 0.00 |  | 5.96 | (5.60-6.31) | 0.00 |  |
|  |  | 2020 | 5.82 | (5.04-6.60) | 0.06 | (-0.71 - 0.83) | 5.70 | (5.09-6.31) | -0.25 | (-1.92 - 0.51) |
|  |  | 2019 | 5.63 | (5.17-6.09) | -0.13 | (-0.80 - 0.54) | 5.87 | (5.28-6.47) | -0.08 | (-0.78 - 0.62) |
|  |  | 2018 | 6.25 | (5.35-7.14) | 0.49 | (-0.33 - 1.30) | 6.40 | (5.65-7.16) | 0.45 | (-0.31 - 1.21) |
|  |  | 2017 | 5.41 | (5.01-5.80) | -0.35 | (-1.02 - 0.31) | 5.61 | (5.12-6.09) | -0.35 | (-1.03 - 0.33) |
|  | Injuries | Mean 2017-19 | 2.93 | (2.78-3.09) | 0.00 |  | 2.83 | (2.69-2.97) | 0.00 |  |
|  |  | 2020 | 2.83 | (2.60-3.06) | -0.10 | (-0.40 - 0.19) | 2.85 | (2.61-3.09) | 0.02 | (-0.25 - 0.29) |
|  |  | 2019 | 3.00 | (3.72-3.27) | 0.06 | (-0.25 - 0.37) | 2.86 | (2.61-3.10) | 0.03 | (-0.24 - 0.31) |
|  |  | 2018 | 2.95 | (2.68-3.22) | 0.01 | (-0.29 - 0.32) | 2.70 | (2.47-2.92) | -0.13 | (-0.40 - 0.14) |
|  |  | 2017 | 2.86 | (2.61-3.11) | -0.08 | (-0.38 - 0.22) | 2.89 | (2.69-3.17) | 0.10 | (-0.18 - 0.37) |

***Table S9:* Number of weekly readmissions in the diagnosis categories. Absolute difference (AD) and relative risk (RR) is calculated with the mean of years 2017-19 as the reference, separately for the weekly period preceding the first wave (week 2-10) and the during first wave of Covid-19 (week 12-22).**

|  |  |  | **Week 2-10** | | | | | | **Week 12-22** | | | | | |
| --- | --- | --- | --- | --- | --- | --- | --- | --- | --- | --- | --- | --- | --- | --- |
| **Diagnosis group** | **Diagnosis category** | **Year** | **No. of readmissions** | **Lowest-highest** | **AD** | **CI (95%)** | **RR** | **CI (95%)** | **No. of readmissions** | **Lowest-highest** | **AD** | **CI (95%)** | **RR** | **CI (95%)** |
| Unaffected by lockdown | Acute myocardial infarction | Mean 2017-19 | 22 | (9-43) | 0 |  | 1.00 |  | 22 | (15-32) | 0 |  | 1.00 |  |
|  |  | 2020 | 19 | (10-30) | -3 | (-7 - 0) | 0.86 | (0.72-1.02) | 13 | (6-20) | -9 | (-11 - -6) | 0.60 | (0.49-0.71) |
|  |  | 2019 | 24 | (18-43) | 2 | (-2 - 6) | 1.09 | (0.93-1.28) | 22 | (15-32) | 0 | (-3 - 3) | 1.01 | (0.86-1.17) |
|  |  | 2018 | 25 | (16-40) | 3 | (-1 - 6) | 1.12 | (0.96-1.31) | 23 | (15-31) | 1 | (-2 - 4) | 1.05 | (0.91-1.22) |
|  |  | 2017 | 18 | (9-26) | -5 | (-8 - -2) | 0.79 | (0.66-0.94) | 20 | (15-28) | -1 | (-4 - 2) | 0.94 | (0.81-1.10) |
|  | Acute abdominal conditions | Mean 2017-19 | 1 | (0-3) | 0 |  | 1.00 |  | 1 | (0-6) | 0 |  | 1.00 |  |
|  |  | 2020 | 1 | (0-3) | 0 | (-1 - 1) | 0.89 | (0.35-2.01) | 1 | (0-2) | 0 | (-1 - 0) | 0.72 | (0.27-1.69) |
|  |  | 2019 | 2 | (1-3) | 1 | (0 - 1) | 1.56 | (0.75-3.07) | 0 | (0-1) | 0 | (-1 - 0) | 0.52 | (0.16-1.35) |
|  |  | 2018 | 1 | (0-2) | 0 | (-1 - 0) | 0.78 | (0.29-1.83) | 2 | (0-6) | 1 | (0 - 2) | 1.86 | (0.97-3.47) |
|  |  | 2017 | 1 | (0-3) | 0 | (-1 - 0) | 0.67 | (0.23-1.65) | 1 | (0-2) | 0 | (-1 - 0) | 0.62 | (0.21-1.52) |
|  | Cerebrovascular disease | Mean 2017-19 | 3 | (0-9) | 0 |  | 1.00 |  | 5 | (0-9) | 0 |  | 1.00 |  |
|  |  | 2020 | 5 | (2-8) | 2 | (0 - 3) | 1.52 | (1.04-2.18) | 4 | (3-6) | -1 | (-2 - 1) | 0.85 | (0.59-1.20) |
|  |  | 2019 | 3 | (2-6) | 0 | (-1 - 1) | 0.99 | (0.63-1.51) | 5 | (0-8) | 0 | (-1 - 2) | 1.07 | (0.77-1.46) |
|  |  | 2018 | 3 | (0-9) | 0 | (-1 - 1) | 1.02 | (0.66-1.55) | 4 | (2-8) | 0 | (-2 - 1) | 0.97 | (0.69-1.34) |
|  |  | 2017 | 3 | (1-7) | 0 | (-1 - 1) | 0.99 | (0.63-1.51) | 4 | (1-9) | 0 | (-2 - 1) | 0.97 | (0.69-1.34) |
| Affected by lockdown | Infections | Mean 2017-19 | 2 | (0-8) | 0 |  | 1.00 |  | 2 | (0-6) | 0 |  | 1.00 |  |
|  |  | 2020 | 2 | (0-4) | 0 | (-1 - 1) | 0.87 | (0.47-1.55) | 0 | (0-1) | -1 | (-2 - -1) | 0.16 | (0.03-0.50) |
|  |  | 2019 | 3 | (1-8) | 1 | (0 - 2) | 1.36 | (0.81-2.23) | 2 | (0-6) | 0 | (-1 - 1) | 1.04 | (0.58-1.77) |
|  |  | 2018 | 2 | (0-5) | 0 | (-1 - 1) | 0.93 | (0.50-1.62) | 1 | (0-4) | 0 | (-1 - 1) | 0.87 | (0.47-1.55) |
|  |  | 2017 | 1 | (0-4) | -1 | (-2 - 0) | 0.71 | (0.36-1.31) | 2 | (0-4) | 0 | (-1 - 1) | 1.09 | (0.62-1.85) |
|  | Injuries | Mean 2017-19 | 5 | (2-11) | 0 |  | 1.00 |  | 4 | (0-9) | 0 |  | 1.00 |  |
|  |  | 2020 | 5 | (2-11) | 0 | (-2 - 1) | 0.91 | (0.63-1.28) | 4 | (1-7) | 0 | (-2 - 1) | 0.92 | (0.64-1.29) |
|  |  | 2019 | 5 | (2-9) | 0 | (-2 - 2) | 0.97 | (0.68-1.36) | 4 | (2-7) | 0 | (-2 - 1) | 0.98 | (0.69-1.37) |
|  |  | 2018 | 5 | (3-9) | 0 | (-2 - 2) | 1.01 | (0.72-1.41) | 4 | (0-9) | 0 | (-1 - 2) | 1.02 | (0.72-1.42) |
|  |  | 2017 | 5 | (3-11) | 0 | (-2 - 2) | 1.01 | (0.72-1.41) | 4 | (0-8) | 1 | (-1 - 1) | 1.00 | (0.71-1.40) |
